# Development of immune radiotherapy with yttrium by targeting Frizzled homologue 10 (FZD10) in cervical cancer

**DOI:** 10.1016/j.gore.2025.101736

**Published:** 2025-04-17

**Authors:** Yuki Okuma, Yuji Ikeda, Osamu Kobayashi, Saki Kamata, Erina Matsuda, Takahiro Nakajima, Keisuke Saito, Naoko Tomita, Yoko Nakanishi, Yosuke Harada, Suyoun Chung, Jae-Hyun Park, Shinobu Masuda, Yusuke Nakamura, Kei Kawana

**Affiliations:** a Department of Obstetrics and Gynecology, Nihon University School of Medicine, 30-1 Oyaguchi-kamimachi, Itabashi-ku Tokyo 173-8610, Japan; b Department of Pathology and Microbiology, Nihon University School of Medicine, 30-1 Oyaguchi-kamimachi, Itabashi-ku Tokyo 173-8610, Japan; c OncoTherapy Science Inc., Kawasaki, Kanagawa 210-0005, Japan; d National Institutes of Biomedical Innovation, Health and Nutrition, Osaka 567-0085, Japan

**Keywords:** Cervical Cancer, Radiotherapy, Immunotherapy

## Abstract

**Objective:**

Radioimmunotherapy (RIT) is a combination of the therapeutic properties of radioisotopes and specific antibodies used to eliminate tumors. ^90^Y-OTSA101 was developed by labeling Frizzled homologue 10 (FZD10) antibody with Yttrium-^90^ (^90^Y). Because cervical cancer exhibits good radiosensitivity, the present study aimed to assess for prevalence of FZD10 in cervical cancer tissue and investigate the potential of ^90^Y-OTSA101 treatment for cervical cancer.

**Methods:**

The expression of FZD10 was evaluated via immunohistochemistry using formalin-fixed paraffin blocks collected from 84 patients with cervical cancer who underwent surgery. Normal cervical from 10 patients, normal ovarian tissues from 10, uterine carcinomas from 10, and uterine sarcomas from 9 were used for comparison.

Mice inoculated with the SiHa cell line, which is derived from cervical squamous cell carcinoma, were used to examine the effects of ^90^Y-OTSA101.

**Results:**

The evaluation results indicated that 70 (91 %) of the cervical cancer cases expressed FZD10 more frequently than 3 (30 %), 1 (10 %), 2 (20 %), and 0 (0 %) of the normal cervix, normal ovary, uterine carcinoma, and uterine sarcoma cases, respectively. The group treated with 3.7 MBq of ^90^Y-OTSA101 demonstrated greater antitumor effects, defined as tumor shrinkage or growth suppression, than the group treated with 1.85 MBq. No significant difference in body weight was observed between the groups during the observation period.

**Conclusions:**

FZD10 may be an appropriate target and ^90^Y-OTSA101 may have potential for the treatment of cervical cancer.

## Highlights

• ・FZD10 was strongly expressed in cervical cancer.
• ・FZD10 is a potential target for cervical cancer treatment.
• ・^90^Y-OTSA101 may have potential for cervical cancer treatment.

## 1 Objective

Cervical cancer remains to be the second leading cause of cancer death among women aged 20 to 39 years in the United States (Siegel et al., 2022). Each year, there are approximately 600,000 new cases of and 350,000 deaths from cervical cancer worldwide (Sung et al., 2020). One of the most effective therapeutics for cervical cancer is radiotherapy, which is widely used as a standard treatment. A randomized trial conducted by Landoni et al., which compared radical surgery and radiotherapy in patients with stage Ib-IIa cervical cancer, revealed no significant differences between the two in terms of 5-year survival and recurrences (Landoni et al., 1997).

Although the range of indications for immune checkpoint inhibitors for cervical cancer has expanded in recent years, local treatment, such as radiation and surgery, is an extremely effective treatment for isolated tumors. However, it is not indicated for multiple metastases or lung lesions that are difficult to treat with radiotherapy. Consequently, the median overall survival of recurrent cervical cancer remains to be 12.0 months, and the development of novel therapeutics is warranted (Tewari et al., 2022).

Radioimmunotherapy (RIT) is a combination of the therapeutic properties of radioisotopes and specific antibodies used to eliminate tumors (Köhler and Milstein, 1975). The antibody delivers the radioisotope to the tumor site where the antigen is expressed, and the radiation can exert its effect on the cell expressing the antigen. By selecting antibodies against tumor-specific antigens and attaching radioisotopes, radiotherapy can be adapted from systemic to local therapy. This concept has been practically applied in B-cell lymphoma. Rituximab is widely utilized to treat B-cell lymphoma; it exerts its antitumor effect by binding to a protein called CD20 that exists on the B-cell surface (Chugh et al., 2015). Yttrium (^90^Y)-ibritumomab tiuxetan is a type of antibody drug that also binds to the CD20 antigen. However, it contains a radioisotope (^90^Y) that emits beta rays. It has achieved a final complete response rate of 87 % in patients with advanced follicular lymphoma (Morschhauser et al., 2008). This result indicates the clinical significance and potential of RIT in cancer therapeutics.

RIT offers several advantages for tumors with heterogeneous antigen expression, the most notable being its ability to kill tumor cells adjacent to those bound by radioimmunoconjugates (the cross-fire effect). This allows RIT to exert cytotoxic effects on tumor cells that are either inaccessible to the antibody or lack sufficient target antigen expression for antibody binding. (White, 2004).

Frizzled homologue 10 (FZD10) was found to be specifically upregulated at extremely high levels in soft tissue sarcomas when analyzed for gene expression (Nagayama et al., 2002). FZD10 is a member of the Frizzled family of seven-transmembrane receptors, cell-surface receptors activated by Wnt proteins and involved in cell function regulation (Hutchings et al., 2017). Genome-wide analysis of gene expressions using a cDNA microarray revealed high FZD10 expression in synovial sarcomas but near-absent expression in the remaining normal adult tissues, except for the placenta (Nagayama et al., 2002). Moreover, previous studies have reported extremely low or no FZD10 protein expression in normal organs, except for the placenta, suggesting that FZD10-targeted therapeutics have limited adverse effects on normal cells and tissues (Nagayama et al., 2005). Although the function of FZD10 remains unclear, some recent studies have reported that its expression is correlated with the advanced stages of gastric and colorectal cancer and that FZD10 may act as a messenger for cancer activation in cells (Scavo et al., 2019, Scavo et al., 2019).

Another study reported that FZD10-mRNA delivering exosomes may be potential messengers of cancer reactivation and play an active role in long-distance metastasis (Scavo et al., 2019). Notably, FZD10-mRNA was reported to be highly expressed in cervical cancer cell lines (Koike et al., 1999).

OTSA101 is a monoclonal antibody that targets the human FZD10 protein. Due to target specificity, OTSA101 was labeled with ttrium-^90^ (^90^Y), a highly energetic beta emitter radioisotope, to develop as RIT. At present, ^90^Y-OTSA101 is being developed for synovial sarcoma; it exhibited stable disease in 3/8 patients with synovial sarcomas in a first-in-human phase 1 trial (Giraudet et al., 2018).

This study aimed to investigate the clinical potential of ^90^Y-OTSA101 in cervical cancer. In detail, we evaluated i) FZD10 expression in cervical cancer and other normal tissues as well as ii) the effect of ^90^Y-OTSA101 *in vivo*.

## 2 Methods

### 2.1 Immunohistochemistry

Four-micrometer sections were prepared from formalin-fixed paraffin-embedded blocks of clinical specimens, including 10 normal cervical tissues, 10 normal ovarian tissues, 10 endometrial carcinomas, and 9 uterine sarcomas. All specimens were obtained with the approval of the ethics committee of Nihon University Itabashi Hospital (RK-170711–6) and written informed consent from the patients. As for cervical cancer specimens, a tissue microarray was established for 77 samples and examined. The sections were deparaffinized, rehydrated, and antigen-activated (pH 9.0, 100 °C, 30 min). An anti-FZD10 antibody (provided by OncoTherapy Science Inc.) was utilized as the primary antibody and Histofine Simple Stain MAX PO (Nichirei, Tokyo) as the secondary antibody; the reactants were visualized in hydrogen peroxide-added DAB solution and contrast-stained with hematoxylin. SYO-1 cells, a synovial sarcoma cell line, was utilized as positive control, whereas LoVo colon adenocarcinoma cells, a cell line that has been shown not to express FZD10, were used as negative control. Positivity was defined as staining greater than 10 % of the total tissue.

### 2.2 Flow cytometry

The protein expression of FZD10 on the cell surface was evaluated in three samples of cervical squamous cell carcinoma tissues collected via surgery at our hospital. The tumor tissues were collected in DMEM (Fujifilm Wako Pure Chemicals Corporation) supplemented with 10 % FBS + 1X Pen/Strep. Cell isolation was performed using a combination of mechanical disruption with scissors and enzymatic disruption with collagenase IV (Gibco), followed by cell isolation using a 70-µm cell strainer. The isolated cells were collected and reacted with primary and secondary antibodies and analyzed within 24 h using BD FACSVerse (BD Bioscience) and the FlowJo software version 10.0.7r2 (Tree Star, Inc., Ashland, OP, USA). A14, a thyroid cancer cell line engineered with a high FZD10 expression, was kindly provided by OncoTherapy Science, Inc. LNCap, a prostate cancer cell line, was obtained from Riken Cell Bank (Ibaraki, Japan).

### 2.3 Real-time PCR

The mRNA expression of *FZD10* in the cell lines was evaluated via real-time PCR. The total RNA extracted from SiHa, HeLa, Caski, and C33A cervical cancer cell lines as well as LNCap prostate cancer cell line, a cell line that has been shown not to express FZD10, as negative control were measured using an absorbance plate reader (Thermo Fisher Scientific). After synthesis of complementary DNA (cDNA), real-time PCR analysis was conducted using Applied Biosystems 7500 Standard Real-Time PCR System (Applied Biosystems). For the *FZD10* expression, HS00273077_s1 (Thermo Fisher Scientific) was used as primer set and β-actin TaqMan Gene Expression Assays (Applied Biosystems, Massachusetts) as endogenous control.

### 2.4 *In vivo* evaluation

All animal experiments were conducted in accordance with the approval of the Animal Experiment Committee of the School of Medicine, conforming to the internal rules for the operation of animal experiments at Nihon University (animal experiment number: AP19MED061-2).

A total of 24 BALB-c nu/nu (female, 4 weeks) mice were purchased (Sankyo Lab Service, Tokyo, Japan) and kept under sterile conditions. They were supplied with sterile food and water *ad libitum*. At 5 weeks of age, 0.1 mL of PBS containing 5 × 10^6^ SiHa cells was subcutaneously injected into the left abdomen of mice, and tumor growth was monitored.

### 2.5 Antibody radiolabeling

A humanized chimeric anti-FZD10 antibody (OTSA101) was kindly provided by OncoTherapy Science, Inc. This antibody was mixed with 1-M sodium acetate and ^90^Y-Cl solution (Perkin Elmer Japan Co., Ltd.) and allowed to react for 5 min, followed by mixing with a DTPA solution that completely chelates the free yttrium after the reaction. The labeling rate was confirmed by measuring the dose three times using a liquid scintillation counter.

### 2.6 Evaluation of radioimmunotherapy with ^90^Y-OTSA101

Three weeks after cell transplantation, the mice were randomly divided into four groups (n = 5 per group) and 1 mL of the PBS solution containing PBS only, OTSA101 alone, ^90^Y alone (1.85 MBq), and ^90^Y-OTSA-101 (1.85 MBq), respectively, were intravenously administered into the tail vein of the mice. ^90^Y dose, 1.85 MBq, was determined from a previous study {Sudo, 2019 #1}. Weekly measurements were performed using calipers to monitor the tumor volume trends and calculated using the formula volume (mm^3^) = (long diameter) × (short diameter)^2^ /2. Relative tumor volume was calculated as the weekly measured volume divided by the volume before the treatment start. Simultaneously, the mice were weighed on a scale. From the viewpoint of humane endpoints, the observation period was set up until the 6th week after the reagent administration.

### 2.7 Evaluation of an irradiation dose with ^90^Y-OTSA101

Two weeks after cell transplantation, the mice were randomly divided into three groups (n = 7 per group), and 1 mL of the PBS solution containing PBS only, ^90^Y-OTSA-101 (1.85 MBq), and ^90^Y-OTSA-101 (3.7 MBq) was intravenously administered into the tail vein of mice, respectively. Thereafter, the same procedure for weekly measurement was followed, as described in the previous paragraph.

### 2.8 Statistical analysis

Comparisons between the groups were made using the Kruskal–Wallis test and the Steel method, with a *P* value < 0.05 indicating a significant difference.

### 2.9 FZD10 expression in cervical cancer specimens and other tissue samples

First, we mainly examined the mRNA expression of *FZD10* in cervical cancer using an online database and found a remarkably higher expression in cervical cancer samples than the other specimens, such as breast and colorectal specimens, respectively (Supplementary Fig. S1). Based on these findings, we further examined the FZD10 protein expression using surgical samples. A total of 116 samples, including 77 from cervical cancer, 10 from normal cervix, 10 from normal ovary, 10 from endometrial cancer, and 9 from uterine sarcoma cases, were examined via immunohistochemical staining. The background characteristics of these samples are presented in Table 1. Among the 77 cervical cancer cases, 55 were squamous cell carcinoma and 60 were Stage I. As surgery is not indicated for previous Stage III or higher, advanced-stage cases were excluded from the analysis. Consequently, FZD10 was frequently expressed in 70 of 77 (91 %) cervical cancer cases but in only 3 of 10 (30 %) normal uterus cases, 1 of 10 (10 %) normal ovary cases, 2 of 10 (20 %) uterine cancer cases, and 0 of 9 (0 %) uterine sarcoma cases (Table 2). Furthermore, high expression (≥50 % positive cells with strong intensity) of FZD10 was found in 41 (53 %) cervical cancer samples (Supplementary Fig. S2), whereas normal uterine tissue and uterine malignancy specimens exhibited no detectable high expression (Supplementary Figs. S2 and 3). These results indicate that targeting FZD10 may be appropriate in cervical cancer treatment.

**Fig. 1:**
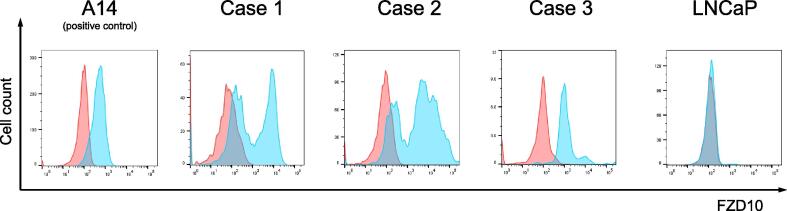
Evaluation of FZD10 expression in cervical cancer FZD10 expression on the plasma membrane was identified in three surgical specimens of cervical cancer. A14 was used as a positive control with high FZD10 expression. Red: Unstained; Blue: FZD10. (For interpretation of the references to colour in this figure legend, the reader is referred to the web version of this article.)

**Table 1:** Characteristics of patients assessed for FZD10 expression.

	cervical cancer (n = 77)	Normal cervix Normal ovary (n = 10)	Endometrial carcinoma (n = 10)	Uterine sarcoma (n = 9)
Age(Median)	45	52.5	53.5	61
Histological subtype				
Squamous cell carcinoma	55	−	−	−
Endometrioid carcinoma	13	−	7	−
Mucus adenocarcinoma	5	−	−	−
Serous adenocarcinoma	1	−	3	−
Neuroendocrine tumors	3	−	−	−
Carcinosarcoma	−	−	−	6
Endometrial stromal sarcoma	−	−	−	3
uterine myoma	−	10	−	−
Stage				
Ⅰ	60	−	1	8
Ⅱ	17	−	3	−
Ⅲ	−	−	4	1
Ⅳ	−	−	2	−

**Table 2:** Positive rates of FZD10 in immunohistochemical staining.

Histological subtype	Positive for FZD10
Cervical cancer	70/77 (91 %)
Normal cervix	3/10 (30 %)
Normal ovary	1/10 (10 %)
Endometrial cancer	2/10 (20 %)
Uterine sarcoma	0/9 (0 %)

**Fig. 2:**
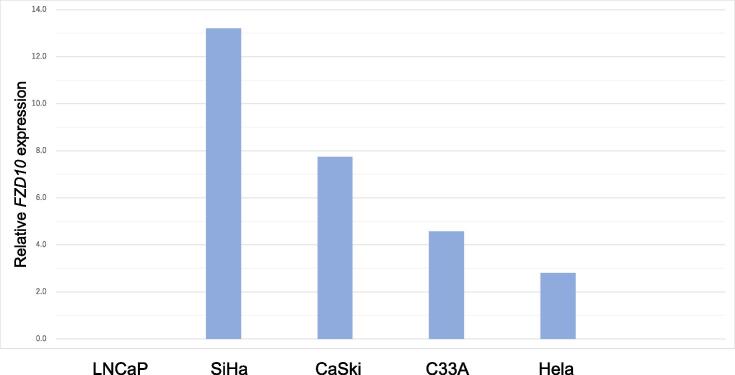
Relative mRNA expression level in cervical cancer cell lines FZD10 expression was examined in cervical cancer cell lines SiHa, Caski, C33A, and HeLa. The differences in its expression levels were compared with those of the prostate cancer cell line LNCap.

**Fig. 3:**
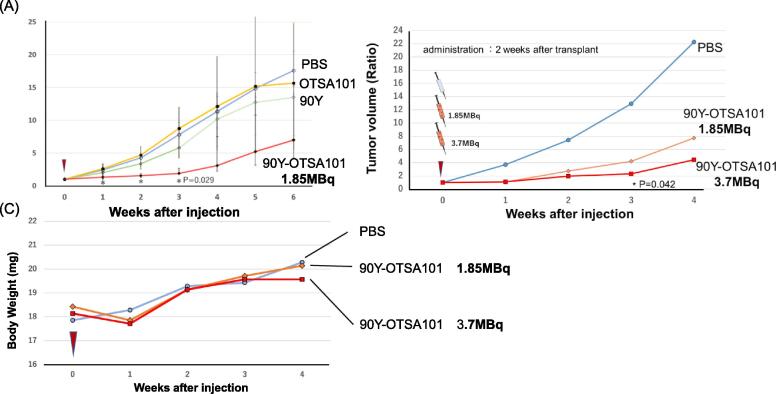
In vivo evaluation of ^90^Y-OTSA101 Using mice subcutaneously implanted with SiHa cell lines, the effects of ^90^Y-0TSA101 were examined. (A) Comparison of ^90^Y-OTSA101 supplemented with PBS, OTSA101 alone, and yttrium alone. (B) Comparison of 1.85 and 3.7 MBq of ^90^Y-0TSA101. (C) Weight variations of mice over time during the experiment.

### 2.10 Expression of FZD10 on the cell surface and selection of cell lines for *in vivo* experiment

Next, we examined the therapeutic potential of targeting FZD10. Considering the mechanism of action as an antibody drug, we determined whether FZD10 was expressed on the cell membrane surface in cervical cancer. Therefore, we evaluated the FZD10 expression on the cell surface using fresh surgical specimens of cervical cancer. As a result, FZD10 expression on the cell surface was observed in 3 of 3 cases (100 %), as illustrated in Fig. 1. Based on these results, antibody drugs targeting FZD10 may be effective for cervical cancer. We also examined the FZD10 expression in human cancer cell lines (Fig. 2). The SiHa cell line exhibited the highest FZD10 expression among the four cervical cancer cell lines examined in this study, whereas LNCap, the negative control, showed no expression.

## 3 *In vivo* evaluation of ^90^Y-OTSA101

After constructing a mouse carcinoma-bearing model with the SiHa cell line, the antitumor effect of **^90^**Y-OTSA101 was examined. All the labeling rates between yttrium-90 and the FZD10 antibody were greater than 96 % (Sup. Table S1). The *in vivo* study results are illustrated in Fig. 3A. No significant difference was observed in the antitumor effects of PBS, OTSA101, or **^90^**Y alone. However, the **^90^**Y-OTSA101-treated group showed significantly reduced tumor size (*P* = 0.029) at 6 weeks after injection. Next, the tumor shrinkage of **^90^**Y-OTSA101 was evaluated at different doses of **^90^**Y. Tumors irradiated with 3.7 MBq of **^90^**Y-OTSA101 shrank more than those irradiated with 1.85 MBq, and analysis including the PBS control group revealed a significant difference (Fig. 3B); however, there were no significant differences in weight loss among the three groups (Fig. 3C).

### 3.1 Tumor cell proliferation after 90Y-OTSA101 treatment

**Fig. 4:**
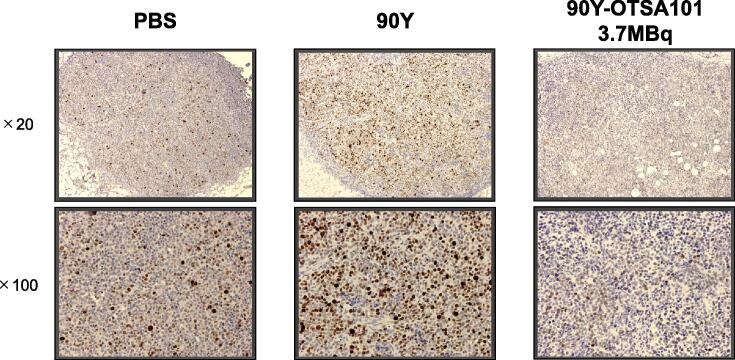
Cell proliferation status after ^90^Y-OTSA101 treatment2 days after ^90^Y-OTSA101 administration, the tumors were removed and immunostained with Ki67 to evaluate the status of cell proliferation.

Finally, Ki67 expression was evaluated to explore cell proliferation in the tumor at 2 days after **^90^**Y-OTSA101 injection. As illustrated in Fig. 4, the Ki67 staining level was lower in the tumor treated with **^90^**Y-OTSA101 than in those treated with PBS and **^90^**Y alone. In particular, the number of cells stained with Ki67 was low in the tumor treated with **^90^**Y-OTSA101. These findings indicate that cell proliferation may be suppressed after **^90^**Y-OTSA101 treatment.

## 4 Discussion

This innovative study applied radiotherapy from local treatment into systemic therapy to the cervical cancer, which is one of the most radiosensitive cancers worldwide (Qin et al., 2014). In this study, we evaluated i) the FZD10 expression in cervical cancer and ii) the effectiveness of **^90^**Y-OTSA101 for cervical cancer.

The selection of tumor-specific molecules is crucial in cancer therapy (Waarts et al., 2022). Surgery allows for complete tumor resection, while chemotherapy, targeting cell proliferation, often leads to side effects like hair loss, pancytopenia, and secondary cancer (Boivin, 1990). Radiotherapy similarly affects the entire irradiated area, causing hematologic and gastrointestinal toxicities (Peters et al., 2000). RIT, however, delivers radiation specifically to cancer cells via antibodies attached to radioisotopes. For RIT to be effective, the target antigen must be highly cancer-specific; otherwise, radiation exposure to normal cells would negate its advantage as a localized therapy.

This study found that FZD10 was strongly expressed in cervical cancer. Positive rate by immunohistochemical staining was higher than uterine cancer or sarcoma. Furthermore, 30 % of the normal cervix cases had high FZD10 expression. This study aimed to evaluate FZD10 as a therapeutic target for cervical cancer. The expression of FZD10 in the normal cervix does not diminish its importance as a therapeutic target. Because FZD10 expression was not strongly observed in normal tissues, except for the placenta (Nagayama et al., 2002), targeting FZD10 appeared to be a highly specific therapy for cervical cancer. Furthermore, FZD10 has been implicated in cell proliferation in association with the Wnt pathway (Kolben et al., 2012, Alrefaei et al., 2020), and high FZD10 expression was associated with poor prognosis in colorectal cancer (Scavo et al., 2018). Clinically, only early-stage cases are indicated for surgery in cervical cancer. Therefore, advanced-stage cases were excluded from this study as the collected samples were limited and the prognosis was not analyzed. However, in our analysis, FZD10 was strongly expressed even in early-stage cervical cancer and the cervix. Since the flow cytometry samples were obtained from surgical specimens, differences in FZD10 positivity were observed among specimens. In two cases, a bimodal distribution was observed; however, FZD10 expression on the cell surface was confirmed. Based on these findings, FZD10 was evaluated as a promising therapeutic target for cervical cancer..

*In vivo* studies were conducted to demonstrate the potential of RIT when using FZD10 as a therapeutic target for cervical cancer. **^90^**Y-OTSA101 has been reported to have high therapeutic potential in synovial sarcoma (Giraudet et al., 2018). In our study, the vehicle, the negative control antibody alone, and yttrium-90 alone exerted no apparent effect, but **^90^**Y-OTSA101 induced a clear antitumor effect. Because our aim was to develop a treatment with a stronger effect, doubling of the radiation dose was investigated, and a stronger antitumor effect was observed; however, there was no significant difference in weight loss. Unfortunately, our study did not demonstrate complete tumor remission. Nevertheless, it can be deduced that a stronger antitumor effect can be achieved with a higher radiation dose. The use of different radioisotopes may also be effective. Recently, a study on RIT for synovial sarcoma with a therapeutic agent that conjugates α-emitter actinium (^225^Ac) to an FZD10 antibody was reported, achieving a 60 % complete remission against a mouse model (Sudo et al., 2022). Such a study or an approach involving the use of radiosensitizers may allow for the development of a stronger treatment than ours.

The present study demonstrated the great potential of targeting FZD10 for the treatment of cervical cancer. FZD10 may be an appropriate target, and **^90^**Y-OTSA101 may exhibit potential for the treatment of cervical cancer. Based on our results, we expect that **^90^**Y-OTSA101 will advance to clinical trials for the treatment of cervical cancer.

CRediT authorship contribution statement

Y.H., S.C., and J.P. are employees of OncoTherapy Science, Inc. Y.N. is a stockholder of OncoTherapy Science, Inc. The other authors declare no competing interests.

Funding Statement

The present work was supported by JSPS KAKENHI Grant Number JP24K12610 and JP23K08878.

## CRediT authorship contribution statement

**Yuki Okuma:** Writing – original draft, Visualization, Validation, Project administration, Methodology, Investigation, Formal analysis, Conceptualization. **Yuji Ikeda:** Supervision, Methodology, Funding acquisition, Data curation, Conceptualization. **Osamu Kobayashi:** Validation, Investigation. **Saki Kamata:** Investigation. **Erina Matsuda:** Investigation. **Takahiro Nakajima:** Investigation. **Keisuke Saito:** Supervision. **Naoko Tomita:** Resources, Project administration, Investigation, Funding acquisition, Data curation. **Yoko Nakanishi:** Validation, Supervision, Resources, Methodology, Investigation. **Yosuke Harada:** Resources, Investigation. **Suyoun Chung:** Resources. **Jae-Hyun Park:** Resources. **Shinobu Masuda:** Writing – review & editing, Supervision. **Yusuke Nakamura:** Supervision. **Kei Kawana:** Writing – review & editing, Supervision, Project administration, Methodology, Funding acquisition, Conceptualization.

## Declaration of competing interest

The authors declare the following financial interests/personal relationships which may be considered as potential competing interests: [Y.H., S.C., and J.P. are employees of OncoTherapy Science, Inc. Y.N. is a stockholder of OncoTherapy Science, Inc. The other authors declare no competing interests.].
