# Supplementary material for: Development of immune radiotherapy with yttrium by targeting Frizzled homologue 10 (FZD10) in cervical cancer
Source: Gynecol Oncol Rep. 2025 Apr 17;59:101736. doi: 10.1016/j.gore.2025.101736 (PMC12433755; doi:10.1016/j.gore.2025.101736)

Supplementary Table 1. Yttrium labeling rate with antibody reagents

	Count	Labeled rate	Average	S.D
BG	1212			
Test paper A1	647381	96.7%	96.6%	0.2%
Test paper A2	22996			
Test paper B1	653670	96.7%		
Test paper B2	23233			
Test paper C1	742011	96.4%		
Test paper C2	28780			

Supplementary Figure 1. Evaluation of FZD10mRNA expression using public online database

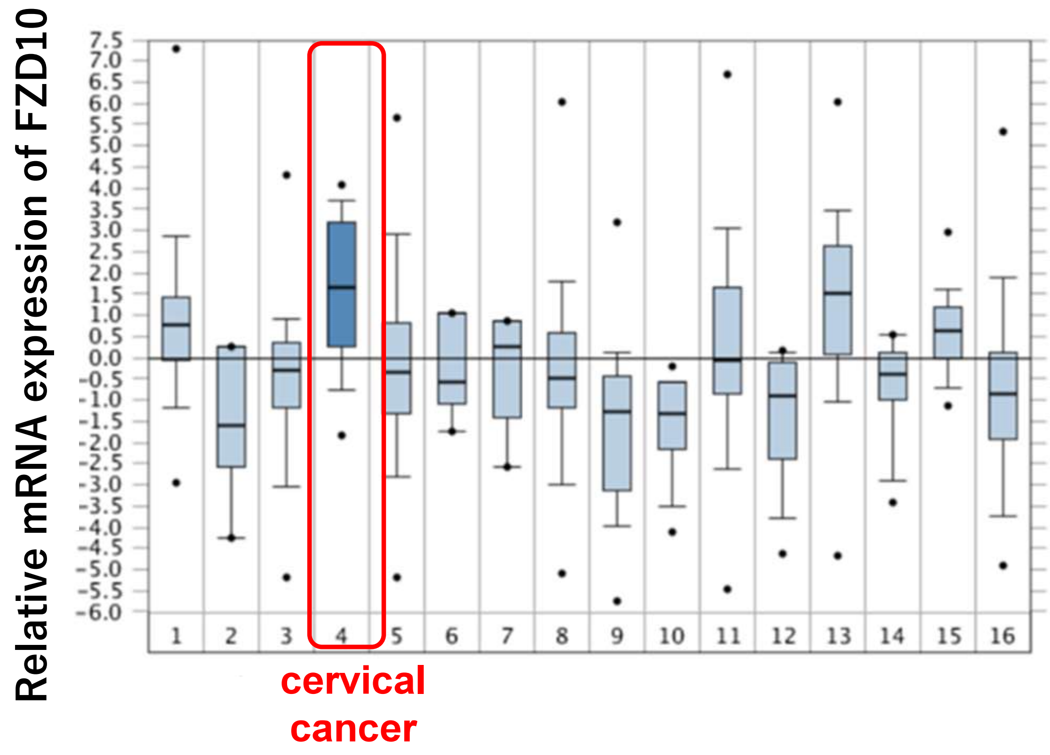

Legend

1. Bladder Cancer (32)
2. Brain and CNS Cancer (4)
3. Breast Cancer (328)
4. Cervical Cancer (35)
5. Colorectal Cancer (330)
6. Esophageal Cancer (7)
7. Gastric Cancer (7)
8. Head and Neck Cancer (41)
9. Kidney Cancer (254)
10. Liver Cancer (11)
11. Lung Cancer (107)
12. Lymphoma (19)
13. Ovarian Cancer (166)
14. Pancreatic Cancer (19)
15. Prostate Cancer (59)
16. Sarcoma (49)

(https://www.oncomine.org/resource/login.html)

Supplementary Figure 2. Immunohistochemical staining of cervical cancer

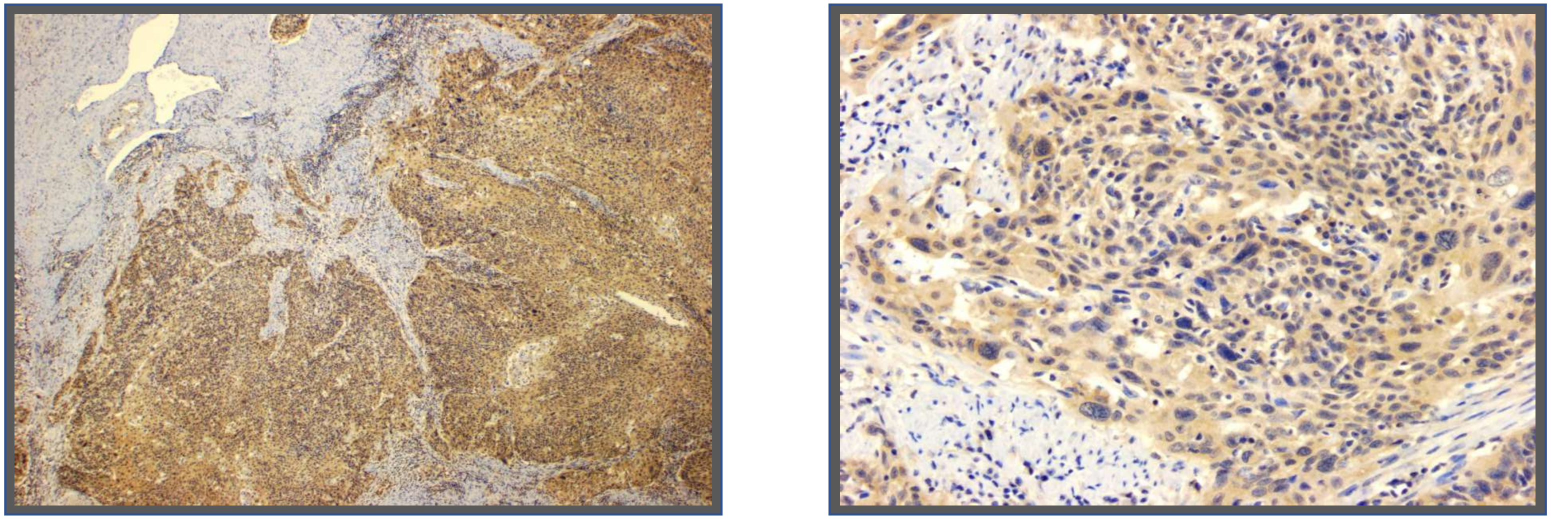

Supplementary Figure 3. Immunohistochemical staining of each organ

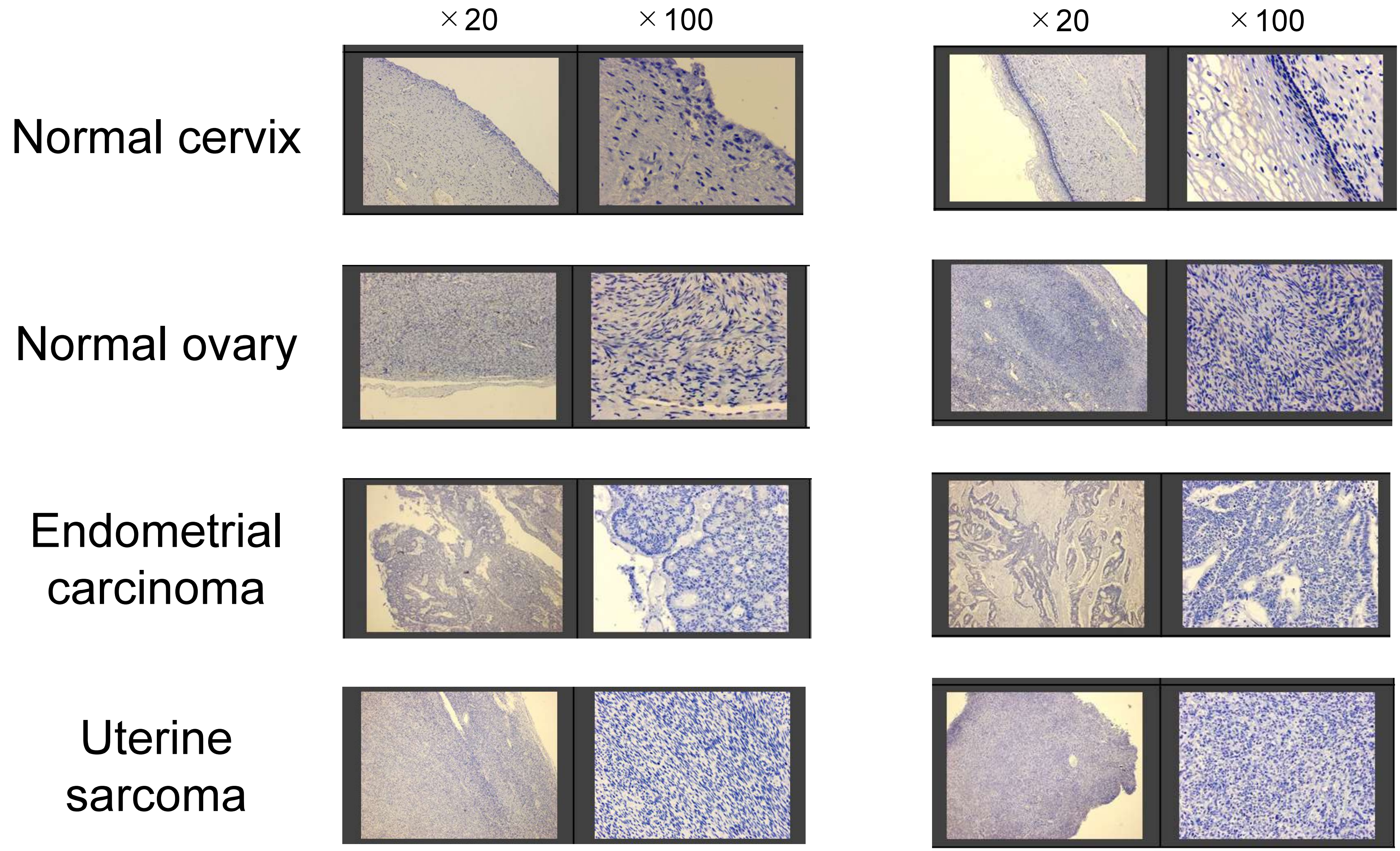

Supplement: Supplementary Data 1 [file mmc1.pdf]
